# Mediators of the stimulatory effect of S1P on colonic Na^+^/K^+^ ATPase

**DOI:** 10.1371/journal.pone.0330818

**Published:** 2025-08-26

**Authors:** Maysoon Noureddine, Sawsan Kreydiyyeh

**Affiliations:** Department of Biology, Faculty of Arts & Sciences, American University of Beirut, Beirut, Lebanon; Universidade Federal do Rio de Janeiro, BRAZIL

## Abstract

The Na ⁺ /K ⁺ ATPase, commonly known as the Na ⁺ /K⁺ pump, plays a crucial role in colonic sodium and water transport, inducing diarrhea or constipation. Inflammatory bowel disease is often associated with diarrhea and elevated levels of sphingosine-1-phosphate (S1P), suggesting a potential relationship between the pump and S1P. This study investigated the effects of S1P on colonic Na ⁺ /K ⁺ ATPase using Caco-2 cells as a model and the S1P analogue used in the treatment of multiple sclerosis, FTY720P. The pump activity was assessed by measuring the amount of inorganic phosphate released in the presence and absence of ouabain, an ATPase inhibitor. FTY720P induced an inhibition of Na ⁺ /K ⁺ ATPase at 15 minutes that was studied in a previous work. This inhibition shifted however, to stimulation at 2 hours, an effect that was abolished in the presence of JTE-013, an S1PR2 antagonist, and was replicated by CYM5520, an S1PR2 agonist. Further mechanistic exploration revealed that when PKC, NF-κB, COX, PKA, and PI3K enzymes were inhibited, FTY720P no longer influenced Na ⁺ /K ⁺ ATPase activity, indicating their involvement in the signaling cascade. Additional evidence supporting this pathway came from activators of these kinases and exogenous PGE₂, both of which stimulated the pump. The results indicate that FTY720P stimulates Na ⁺ /K ⁺ ATPase at 2 hours by binding to S1PR2, leading to PKC activation, followed by NF-κB-mediated induction of PGE₂ synthesis. PGE₂ then binds to its EP4 receptors, activating PKA and PI3K, ultimately resulting in an increase in the pump’s activity. These findings will open the door to targeted regulation of these intermediate molecules which could potentially alleviate certain undesirable effects of the drug.

## Introduction

Maintaining intestinal ion homeostasis is crucial for proper digestive function. Disruptions in this balance can lead to clinically significant outcomes, most notably diarrhea. In the colon, the Na ⁺ /K ⁺ ATPase plays a central role in regulating fluid and electrolyte absorption, owing to its distinct expression profile and functional attributes.

Electrolyte imbalance is a hallmark of inflammatory bowel disease (IBD), which has seen a sustained global rise in both incidence and prevalence over recent decades [1,2]. Despite growing recognition of this burden, the molecular mechanisms by which inflammatory mediators perturb ion transport processes in IBD remain incompletely characterized. Among the key regulators of epithelial transport affected by inflammation is the Na ⁺ /K ⁺ ATPase, whose pivotal role in colonic electrolyte and water handling is well established. By generating a sodium electrochemical gradient, the ATPase provides the driving force for sodium uptake, which is followed by water movement by osmosis. Changes in its activity significantly impact various colonic transporters. A higher activity increases the sodium gradient and Na⁺ absorption via ENaC and NHE3, promotes Cl⁻ absorption through the coupled Cl ⁻ /HCO₃ ⁻ exchanger (DRA), and reduces the CFTR-mediated Cl⁻ secretion. Conversely, a lower activity reduces the sodium gradient, impairs Na⁺ and Cl⁻ absorption, and increases Cl⁻ secretion, potentially leading to intestinal water retention and diarrhea.

Diarrhea is one of the symptoms of inflammatory bowel disease, a disease that is characterized by chronic intestinal inflammation that disrupts epithelial barrier integrity and impairs nutrient and electrolyte transport [3]. The degree of inflammation was reported to correlate negatively with the activity of colonic Na^+^/K^+^ ATPase [4]. Another alteration observed in IBD is an elevation in sphingosine-1-phosphate (S1P) levels in inflamed colonic tissues, which has been attributed to increased expression of sphingosine kinase [5,6]. These elevated S1P levels promote lymphocyte infiltration into the gut mucosa and amplify inflammatory cascades compromising the function of ion transporters such as NHE3 and DRA [7,8], leading to defective Na⁺ and Cl⁻ absorption and resulting in electrolyte imbalances and diarrhea. Moreover, S1P signaling has been implicated in modulating tight junction proteins, further exacerbating barrier dysfunction and fluid loss [9]. Together, these mechanisms highlight a complex interplay where S1P-driven inflammation not only perpetuates mucosal damage but also contributes to the electrolyte disturbances observed in IBD patients.

The concurrent changes in S1P levels and Na ⁺ /K ⁺ ATPase activity in IBD suggest a possible mechanistic interplay that warrants further investigation. Such an interplay was confirmed in a previous study showing that S1P mediates the early downregulatory effect of TNF-α on Na ⁺ /K ⁺ ATPase activity in HepG2 cells [10].

Building upon these observations in hepatic cells, our earlier work in colonic Caco-2 cells demonstrated a similar inhibitory effect of the S1P agonist FTY720P on the Na^+^/K^+^ ATPase that peaked at 15 minutes [11] and was mediated via S1PR2 and PGE2. However, to maintain homeostasis, negative feedback mechanisms are typically activated in an attempt to restore the electrolyte and water balance necessitating a return in the activity of the ATPase back to normal levels. Although this compensatory response is highly anticipated, the temporal dynamics of S1P-mediated regulation of the Na ⁺ /K ⁺ ATPase remain unexplored. Whether the effect of S1P or its agonist FTY720P on Na ⁺ /K ⁺ ATPase activity persists or varies at later time points remains an open question that we aim to address in this study. It is worth noting that S1P plays a crucial role in multiple sclerosis (MS) by regulating immune cell migration and inflammation. Its agonist and receptor modulator FTY720P (fingolimod), acts by preventing lymphocyte egress from lymph nodes, thereby reducing immune-mediated attacks on the central nervous system. As a result, FTY720P has received FDA approval for the treatment of MS. However, its effects on gastrointestinal physiology remain insufficiently characterized and represent a still understudied facet of its clinical profile. One such possibility is that FTY720P may alter Na ⁺ /K ⁺ ATPase activity and gut function, potentially contributing to undesirable side effects during MS therapy.

In this study, we propose and test the hypothesis that S1P/FTY720P signaling exerts a biphasic, time-dependent effect on colonic Na ⁺ /K ⁺ ATPase activity mediated by the activation of specific receptors. We further propose that this effect manifests as a transient inhibition followed by adaptive modulation. Our findings will provide insight into dynamic feedback mechanisms governing epithelial ion homeostasis under inflammatory conditions, and help elucidate the transient diarrhea frequently observed in MS patients receiving S1P receptor modulators [12].

This study is the first to investigate the temporal dynamics of FTY720P-induced modulation of Na ⁺ /K ⁺ ATPase activity and to delineate the signaling pathway involved. The presence of such a time-dependent effect will shed light on how the cell response may evolve over time in order to adapt to the physiological or disease states. In addition, identification of the molecules involved in the action of FTY720P on the Na^+^/K^+^ ATPase would inspire targeted drug development for autoimmune diseases affecting the central nervous system and gut functions such as IBD and MS.

## Materials and methods

### Materials

FTY720P and NF-κB inhibitor were purchased from Santa Cruz Biotechnology, CA, USA. Phorbol-12-myrsitate-13-acetate (PMA), Calphostin C, Adenosine-3’,5’-cyclic Monophosphorothioate, Rp-Isomer Triethylammonium salt (RpcAMP), and Wortmannin were bought from Calbiochem, San Diego, USA. Biorad assay and protein reagent, nitrocellulose membranes and western blotting luminol reagent (Clarity western ECL substrate) were obtained from Biorad, California, USA. Prostaglandin (PGE2), indomethacin, Adenosine 5’-triphosphate disodium salt (ATP), ouabain, Dulbecco’s Minimal Essential Medium (DMEM) with 4500 mg/L glucose and pyridoxine HCL, Fetal Bovine Serum (FBS), Penicillin/Streptomycin, 10x Phosphate Buffered Saline (PBS) without magnesium and calcium, and Trypsin-EDTA were bought from Sigma, Chemical Co, St Louis Missouri, USA. L-826266, SC-51089, BGC 20–1531 hydrochloride, butaprost, and PF-04418948 were purchased from Cayman Chemical Company, Michigan, USA. All other chemicals were purchased from Sigma, Chemical Co, St Louis Missouri, USA. Antibodies were purchased from Santa Cruz. The Caco-2 cell line (male human colonic adenocarcinoma cell line, RRID: CVCL_0025) was obtained in 2021 from the American Type Culture Collection (ATCC). Cells were authenticated by ATCC and certified to be mycoplasma-free upon receipt. Later on routine mycoplasma screening was conducted every 6 weeks in our lab.

### Methods

Caco-2 cells, a human epithelial cell line derived from a colorectal adenocarcinoma, were used in this study as a model of colonic cells. The proposed hypothesis was tested using pharmacological inhibitors and time-course experiments. It should be noted however that while Caco-2 studies provide valuable mechanistic insights—particularly into epithelial transport, barrier function, and signaling pathways under controlled conditions—a key limitation stems from their cancerous origin and the possibility that they harbor oncogenic mutations or altered regulatory pathways that diverge from those of normal colonic epithelium

### Caco-2 cell culture

Caco-2 cells at passages 28−50 were seeded in DMEM supplemented with 10% FBS, 1% penicillin (100 µg/ml), and streptomycin (100 µg/ml), on 100 mm culture plates at a density of 120,000 cell/ml, and incubated at 37°C in a humidified incubator (95% O2, 5% CO2). The seeding density was based on previous work [13]. At 85−90% confluence, and following a 12hr-starvation period during which the cells were incubated in DMEM (described above) without FBS, the cells were treated for 2 hours with 7.5 nM FTY720P (the same concentration at which FTY720P modulated the activity of the ATPase in HepG2 cells and in Caco-2 cells at 15 min). At the end of the treatment, the cells were scraped, homogenized in 1.5 mL tubes using a cordless drive unit with polypropylene pestles, suitable for small-volume mechanical disruption (purchased from Thomas Scientific), and spun for 30 min at 20000 g and 4°C. The supernatant was used to assay for Na^+^/K^+^ ATPase activity and for western blot analysis after quantification of the proteins according to the Bradford method.

All treatments and their corresponding controls were run in triplicates. Cell viability was tested by the trypan blue exclusion test and was found not to be affected by FTY720P used at a concentration of 7.5 nM.

### Na^+^/K^+^ ATPase assay

To assay the activity of the Na + /K + ATPase, three aliquots were taken from each treatment replicate and its corresponding control.

The assay was conducted as described by Esmann [14] by measuring the amount of inorganic phosphate liberated in presence and absence of ouabain, a specific inhibitor of the ATPase. Proteins in the supernatant were quantified using the Bradford method and their concentration was adjusted to 0.5 μg/μl with histidine buffer (pH 7.4, 150 mM). Samples were then incubated for 30 min at room temperature with 1% saponin added at a ratio of 1:4, in presence of phosphatase inhibitors (2.7 mM pyrophosphate, 2.7 mM glycerophosphate). Aliquots from each sample were taken in triplicates and were incubated at 37⁰C in histidine buffer containing NaCl (121.5mM), KCl (19.6 mM,), MgCl2 (3.92 mM) under four distinct conditions: (1) with ATP (2.94 mM), to asses total ATPase activity; (2) with ATP and 1 mM ouabain to inhibit Na ⁺ /K ⁺ ATPase and reveal ouabain-insensitive background ATPase activity; (3) without ATP to account for non-enzymatic background signal; and (4) with ouabain but without ATP to confirm the inhibitor alone does not interfere with the assay readout. Ouabain-sensitive activity, representing Na ⁺ /K ⁺ ATPase function, was calculated by subtracting values obtained in the presence of ouabain from total ATPase activity. The no-ATP control was used to correct for baseline absorbance or phosphate release unrelated to ATP hydrolysis. When ouabain or ATP were absent, they were replaced with an equal volume of water. The reaction was stopped by the addition of 50% trichloroacetic acid at a ratio of 1:10 (v/v) and the samples were spun at 3000g for 5 min. The amount of inorganic phosphate liberated in the supernatant was measured colorimetrically at 750 nm according to the method of Taussky & Shorr [15]. The results are reported as a percentage of the control values.

### Caco-2 cells treatment

#### Time response study on the effect of FTY720P on the Na^+^/K^+^ ATPase activity.

After overnight starvation, Caco-2 cells were treated with 7.5 nM FTY720P for different time periods up to 180 minutes. For the control, an equal volume of the vehicle, DMSO, was used. When the cells were treated with FTY720P in the presence of an inhibitor of a signaling molecule, the inhibitor was always added 30 min before FTY720P.

#### Identification of the S1P receptors involved in the effect of FTY720P.

The results of a previous work studying the expression and abundance of S1P receptors in Caco-2 cells revealed that S1PR2 is the most abundantly expressed receptor followed by S1PR3, while S1PR1 is mildly expressed and S1PR4 and S1PR5 failed to be detected [11]. To determine the type of S1P receptor involved in the effect of FTY720P on the Na^+^/K^+^ ATPase, each of the S1PR2s and S1PR3s were blocked with their respective antagonists JTE-013 (1 µM) and CAY10444 (17.4 µM). The antagonists were added 30 min before FTY720P.

For further confirmation, cells were treated for 2hrs with either CYM5520 (2.5 µM) or CYM5541 (2 µM), respective agonists of S1PR2 and S1PR3.

#### Involvement of PGE2 and determination of the EP receptors activated.

The involvement of PGE2 was also assessed by treating Caco-2 cells with indomethacin (100 µM), a COX inhibitor, added 30 min before FTY720P. The effect of exogenous PGE2 (1nM, 2hrs) was also studied.

Agonists and antagonists were used to identify the EP receptors through which PGE2 acts. EP1, EP2, EP3 and EP4 were blocked respectively with SC-1922 (100μM), PF-04418948 (1μm), L-798106 (10 µM) BGC-20–1531-HCl (10 μM) added 30 min before the 2 hour-treatment with PGE2.

#### Involvement of PKA and PKC.

S1P and EP receptors can be coupled to Gi or Gs, affecting cAMP levels and PKA activity. To determine whether PKA is part of the signaling pathway, the effect of FTY720P on the ATPase was studied in the simultaneous presence of RpcAMP (30μM), a PKA inhibitor, added 30 min prior to FTY720P. Cells were also treated for 2 hours with dbcAMP (10 µM), a cell-permeable cAMP analogue.

Since S1P and EP receptors may couple also to Gq which stimulates PLC and PKC, cells were treated with PMA (100nM, 2hrs), a PKC activator, and with FTY720P in the presence of calphostin C (50 nM), a PKC inhibitor.

#### Involvement of calcium and NF-κB.

To determine whether intracellular calcium is necessary as a second messenger for the pathway initiated by FTY720P, Caco-2 cells were treated with BAPTA/AM (20 nM), a calcium chelator, added 30 min before FTY720P.

NF-κB is known to increase the transcription of COX-2 leading to PGE2 synthesis. To check for its involvement, Caco-2 cells were treated with FTY720P in the simultaneous presence of NF-κB inhibitor (10 μg/ml).

#### Involvement of PI3K and ERK.

S1P and PGE2 may activate PI3K and ERK1/2. The involvement of the two kinases was examined by treating the cells with FTY720P or PGE2 in the simultaneous presence of wortmannin (100 nM), an inhibitor of PI3K, or PD98059 (50 µM), an ERK1/2 inhibitor.

#### Positioning the identified signaling molecules with respect to each other.

The position of ERK in the pathway with respect to PGE2, PKA and PI3K, was examined by western blot analysis by determining changes in the protein level of phosphorylated ERK in cells treated with dbcAMP (10 µM, 2 hrs) or with PGE2 (1 nM, 2 hrs) in the simultaneous presence of wortmannin (100 nM).

#### Locating PKC, PKA and PI3K with respect to PGE2.

To determine if PKC acts downstream or upstream of PGE2, Caco-2 cells were treated with the prostaglandin in presence of calphostin (50 nM) or with PMA (100nM) in presence of indomethacin (100 µM), a blocker of PGE2 synthesis.

The location of PKA and PI3K was determined by studying the effect of PGE2 on the Na^+^/K^+^ ATPase in the presence of RpcAMP (PKA inhibitor, 30μM) or wortmannin (100 nM). The direct effect of dbcAMP (10 µM) in the presence of indomethacin (100 µM,) was also studied.

Furthermore, to position PKC with respect to PKA and PI3K, the effect of PMA was studied in the presence of RpcAMP an inhibitor of PKA, or in the presence of wortmannin (100 nM).

#### Western blot analysis.

Forty micrograms proteins from the homogenate were loaded and resolved on a 10% SDS polyacrylamide gel, transferred to a nitrocellulose membrane, and incubated overnight at 4°C with a specific primary monoclonal antibody against p-ERK (cat # sc-7383; used at a dilution of 1:200), ERK (cat # sc-514302 used at a dilution of 1:550), or GAPDH (cat #sc-47724 used at a dilution of 1:150). The membranes were then incubated with HRP-conjugated secondary antibodies (cat # sc-516102 used at a dilution of 1:4000) for 1 hour at room temperature. The signal was detected by chemiluminescence using Clarity ECL Substrate, and its intensity was determined using a ChemiDocTM imaging system. Exposure times were adjusted to avoid signal saturation and remained constant across all lanes to ensure comparability. GAPDH expression was used to check for equal loading. The bands were normalized to GAPDH or to total ERK using Image lab software following the software’s instructions. The linearity of detection was confirmed by linear regression analysis, yielding an R² value ≥ 0.98 across the 60–10 µg range, validating the quantitative reliability of the assay under these conditions.

### Statistical analysis

Data distribution was assessed for normality using the Shapiro–Wilk test. To identify potential outliers, Grubbs’ test was applied at a significance level of α = 0.05. Unless otherwise stated, normally distributed data were analyzed using a one tailed student’s t-test or a one-way analysis of variance, followed by post hoc multiple comparisons adjusted via the Tukey–Kramer method to account for unequal sample sizes.

Results are presented as mean ± standard error of the mean (SEM). A *p*-value < 0.05 was considered statistically significant. All analyses were performed using the GraphPad InStat 3.

## Results

### FTY720P increases Na^+^/K^+^ ATPase activity at 75min and beyond

**Fig 1:**
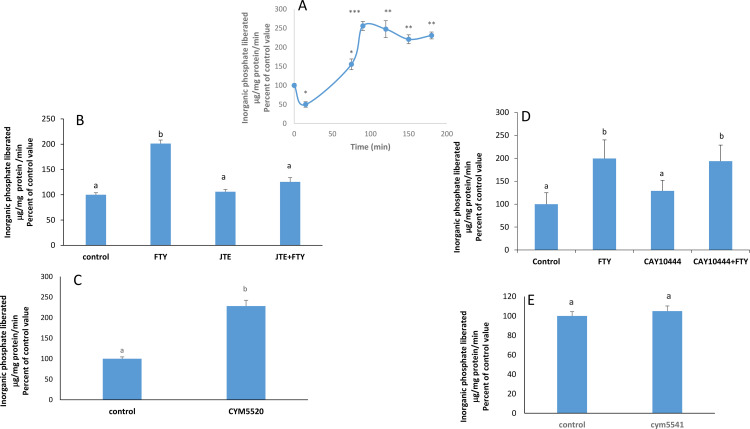
Effect of FTY720P on the Na+/K+ ATPase activity. A) Time-dependent effect of FTY720P (7.5nM) on the activity of Na + /K + ATPase. Values are means ± SE. N = 3. * Significanlty different from the control p < 0.05; **: significantly different from the control p < 0.01; ***: significantly different from the control p < 0.001. Significant differences were tested by ANOVA followed by a Tukey-Kramer test. **B)** Effect of FTY720P (7.5 nM) on the Na + /K + ATPase activity in the presence of JTE-013 (1 µM) at 2 hours. The results are means ± SEM. N = 3. Bars not sharing a common superscript are significantly different from each other at p < 0.01 as revealed by a one-way ANOVA (F = 89.47; p = 0.0001) followed by a Tukey-Kramer test. **C)** Effect of CYM5520 (2.5 µM) on the activity of Na + /K + ATPase at 2 hours. The results are means ± SEM. N = 3. Bars not sharing a common superscript are significantly different from each as indicated by a one-tail Student t-test (p = 0.0019; t = 6.069.45; df = 4). **D)** Effect of FTY720P on Na + /K + ATPase activity in the presence of CAY10444 (17.4 µM). at 2 hours. The results are means ± SEM. N = 3. Statistical significance was tested by a one-way ANOVA (F = 11.59; p = 0.0028) followed by a Tukey-Kramer Multiple Comparisons Test. Bars not sharing a common superscript are significantly different from each other at p < 0.01. **E)** Effect of CYM5541 (2 µM) on the Na + /K + ATPase activity at 2 hours. The results are means ± SEM. N = 3. Bars sharing a common superscript are not significantly different from each other as indicated by a Student t-test (p = 0.337; t = 0.45; df = 4).

FTY720P modulated time-dependently the activity of the Na^+^/K^+^ ATPase in Caco-2 cells. A significant inhibition was observed at 15 min while activation was observed at later time intervals reaching a peak at 90 min (Fig 1A). Consequently, the two-hour time point was chosen as the optimal time for the FTY720P stimulatory action and was adopted in all subsequent experiments. This work focused on the stimulatory effect appearing at 2 hrs.

### FTY720P stimulates the Na^+^/K^+^ ATPase through S1PR2

We showed previously that in Caco-2 cells S1PR2 and S1PR3 are the major receptors present, with S1PR2 having the highest expression [11]. Blocking S1PR2 with the specific antagonist JTE-013 abolished completely the effect of FTY720P and the ATPase activity went back to control values (Fig 1B). Treating Caco-2 cells with the S1PR2 agonist CYM5520 resulted in a significant increase in the activity of the pump (Fig 1C).

Since S1PR3 is also expressed in Caco-2 cells, although at lower levels, its involvement in the effect of FTY720P on the pump was also investigated. In presence of its antagonist CAY 10444, FTY720P was still able to increase the activity of the Na^+^/K^+^ ATPase (Fig 1D) while its agonist CYM5541 had no effect on the pump (Fig 1E).

### PKC is a mediator in the stimulatory pathway initiated by FTY720P

**Fig 2:**
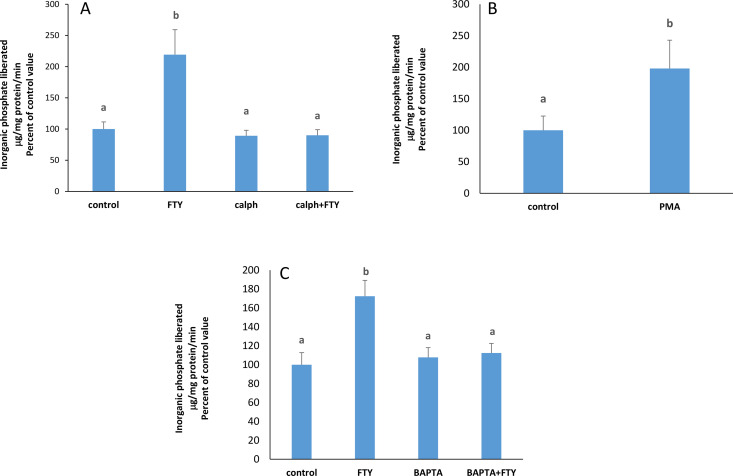
Effect of FTY720P on the Na + /K + ATPase activity in the presence of calphostin (50 nM) at 2 hours. The results are means ± SEM. N = 3. The data was tested for significant differences by a one way- ANOVA (F = 20.27; p = 0.0004) followed by a Tukey-Kramer test. Bars not sharing a common superscript are significantly different from each other at p < 0.0001.**B)** Effect of PMA on the Na + /K + ATPase activity at 2 hours. The results are means ± SEM. N = 3. Bars not sharing a common superscript are significantly different from each other as revealed by Student’s t-test (p = 0.0006; t = 15.021; df = 4). C) Effect of FTY720P on the Na + /K + ATPase activity in the presence of BAPT-AM (20 nM) at 2 hours. The results are means ± SEM. N = 3. Statistical significance was tested by a one-way ANOVA (F = 14.07; p = 0.0015) followed by a Tukey-Kramer test. Bars not sharing a common superscript are significantly different from each other at p < 0.01.

S1PR2 may couple to Gq inducing PLC and PKC activation. The stimulatory effect of FTY720P on the pump did not appear when PKC was inhibited with calphostin C (Fig 2A) while PMA, a PKC activator, exerted a similar stimulatory effect on the ATPase (Fig 2B).

### Activation of the Na^+^/K^+^ ATPase by FTY720P requires calcium

Because some types of PKCs are calcium-dependent, we examined the implication of calcium in the stimulation of the Na^+^/K^+^ ATPase by FTY720P, using the calcium chelator, BAPTA-AM. BAPTA-AM prevented FTY720P from exhibiting its stimulatory effect on the pump (Fig 2C).

### FTY720P induces PGE2 synthesis

**Fig 3:**
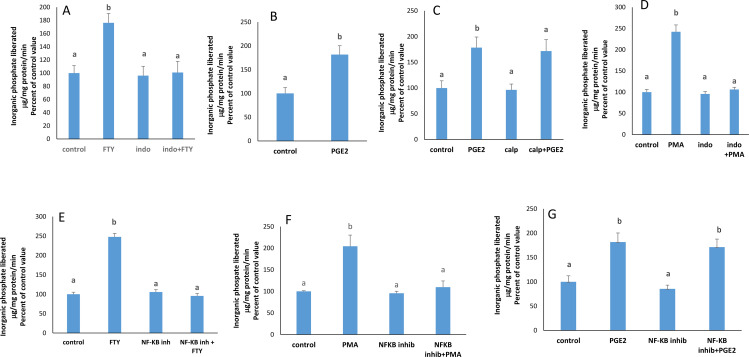
Involvement of PGE2, PKC, and NF- k B. A) Effect of FTY720P on the Na + /K + ATPase activity in the presence of indomethacin (100µ M) at 2 hours. The results are means ± SEM. N = 3. The data were tested for significant differences using a one way-ANOVA (F = 15.29; p = 0.0011) followed by a Tukey-Kramer test. Bars not sharing a common superscript are significantly different from each other at p < 0.001. **B)** Effect of PGE2 (1nM) on the Na + /K + ATPase at 2 hours. The results are means ± SEM. Statistical significance was tested by a Student’s t-test (p = 0.0095; t = 3.809; df = 4). Bars not sharing a common superscript are significantly different from each other. **C)** Effect of PGE2 (1 nM) on the Na + /K + ATPase activity in the presence of calphostin (50 nM) at 2 hours. The results are means ± SEM. N = 3. One- way ANOVA was conducted to test for statistical significance (F = 13.8; p = 0.0084) followed by a Tukey-Kramer test. Bars not sharing a common superscript are significantly different from each other at p < 0.01.**D)** Effect of PMA (100nM) on Na + /K + ATPase activity in the presence of indomethacin (100 µM) at 2 hours. The results are means ± SEM. N = 3. Statistical significance was tested by a one-way ANOVA (F = 12.99; p = 0.0019) followed by a Tukey-Kramer test. Bars not sharing a common superscript are significantly different from each other at p < 0.01. **E)** Effect of FTY720P (7.5 nM), **F)** PMA (100 nM), **G)** PGE2 (1nM) on the activity of Na + /K + ATPase in the presence of NF-κB inhibitor (10 μg/ml) at 2hours. The results are means ± SEM. N = 3. Statistical significance was tested by a one-way ANOVA (FTY: F = : F: 18.61; p = 0.0006- PMA: F = 16.06; p = 0.0010 - PGE2: (F = 10.14; p = 0.0042) followed by a Tukey-Kramer test. Bars not sharing a common superscript are significantly different from each other at p < 0.01.

S1P is known to induce PGE2 synthesis which is catalyzed by the COX enzymes [16]. In presence of indomethacin, a COX enzyme- inhibitor, the effect of FTY720P disappeared completely (Fig 3A). Furthermore, exogenous PGE2 exerted a significant increase in the Na^+^/K^+^ ATPase activity (Fig 3B).

### PKC is upstream PGE2

In the presence of calphostin C, PGE2 could still induce its stimulatory effect on the pump (Fig 3C), while when PGE2 synthesis was blocked with indomethacin, PMA could not exert any effect on the ATPase (Fig 3D).

### NF-κB is along the pathway

NF-κB is a transcription factor known to promote COX-2 expression leading to PGE2 synthesis. When NF-κB was inhibited, the stimulatory effect of FTY720P and PMA was not manifested anymore (Fig 3E & 3F), while PGE2 could still exert its stimulatory effect on the pump (Fig 3G).

### PGE2 acts via EP-4

**Fig 4:**
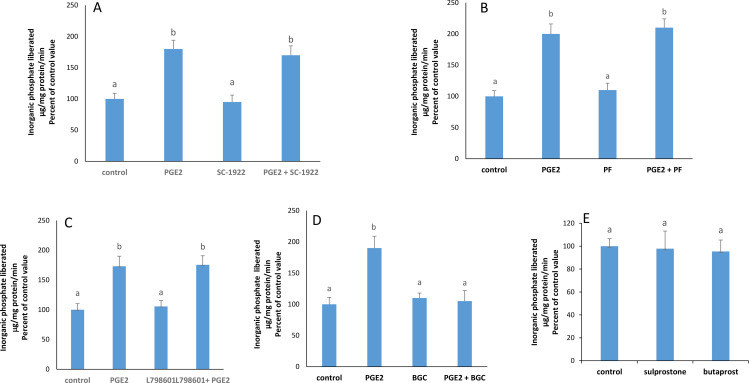
Effect of PGE2 (1nM) on the activity of the Na + /K + ATPase in the presence of the A) EP1 blocker, SC-1922 (100μm) (F: 15.39;p 0.0002); B) EP2 blocker, PF-04418948 (1μ m) (F: 21.21; p:0.0002); C) the EP3 blocker, L-798106 (10M) (F:7.771; p 0.0093). **D)** the EP4 blocker, BGC-20-1531-HCl (10 μM) (F: 7.49; p: 0.0104). **E)** Effect of butaprost and sulprostone on the activity of the Na + /K + ATPase at 2 hours. The results are means ± SEM. N = 3. Statistical significance was tested by a one -way ANOVA (SC-1922: F = 24.33; p = 0.0002 -PF-04418948: F = 20.84; p = 0.0004 - L-798106: F = 15.53; p = 0.0011 – BGC-20-1531-HCl: F = 6.53; p = 0.0194 – butaprost + sulprostone: (F = 0.04075; p = 0.9603) followed by a Tukey-Kramer test. Bars not sharing a common superscript are significantly different from each other at p < 0.01.

The stimulatory effect of PGE2 still appeared in the presence of SC-1922, PF-04418948, and L-798106, respective blockers of EP1, EP2, and EP3 receptors, but disappeared completely when EP4 receptors were blocked with BGC-20–1531 (Fig 4A, 4B, 4C and 4D).

Sulprostone, an EP-1 and EP-3 agonist, as well as butaprost, an EP2 agonist, did not affect the ATPase activity (Fig 4E).

### PKA and PI3K mediate the stimulatory effect of FTY720P on Na^+^/K^+^ ATPase

**Fig 5:**
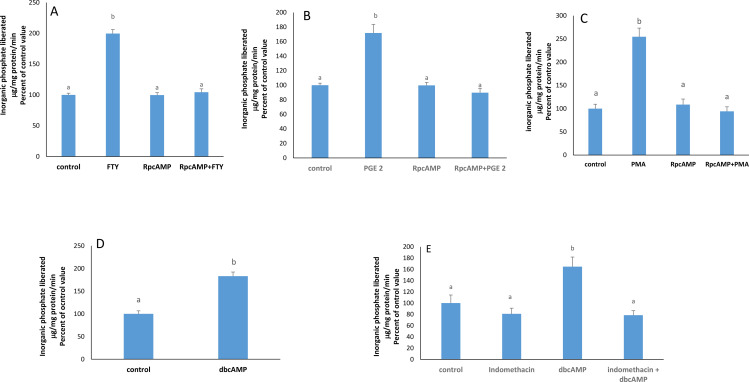
A) Effect of FTY720P on Na + /K + ATPase activity in the presence of RpcAMP (30μ M) at 2 hours. The results are means ± SEM. N = 3. Statistical significance was tested bya one- way ANOVA (F = 35.99; p = 0.0002) followed by a Tukey-Kramer test. Bars not sharing a common superscript are significantly different from each other at p < 0.01. **B)** Effect of PGE2(1nM) on Na + /K + ATPase activity in the presence of RpcAMP (30μM) at 2 hours. The results are means ± SEM. N = 3. One-way ANOVA was conducted to test for statistical significance (F = 17.53; p = 0.0044) followed by a Tukey-Kramer test. Bars not sharing a common superscript are significantly different from each other at p < 0.05.**C)** Effect of PMA (100nM) on Na + /K + ATPase activity in the presence of RpcAMP (30μM) at 2 hours. The results are means ± SEM. N = 3. Statistical significance was tested by a one-way ANOVA (F = 27.12; p < 0.0003) followed by a Tukey-Kramer test. Bars not sharing a common superscript are significantly different from each other at p < 0.01. **D)** Effect of dbcAMP (10 µM) on Na + /K + ATPase activity at 2 hours. The results are means ± SEM. Statistical significance was tested by a Student’s t-test (p = 0.0107; t = 3.672; df = 4) Bars not sharing a common superscript are significantly different from each other.**E)** Effect of dbcAMP (10 µM) on Na + /K + ATPase activity in the presence of indomethacin (100 µM) at 2 hours. The results are means ± SEM. N = 3. One- way ANOVA was conducted to test for statistical significance (F = 9.791; p < 0.0047) followed by a Tukey-Kramer test. Bars not sharing a common superscript are significantly different from each other at p < 0.01.

EP4 receptors are known to signal via PKA and PI3K. The stimulatory effect of FTY720P, PGE2, and PMA on the pump did not appear in the presence of RpcAMP, a PKA inhibitor (Figs 5A, 5B and 5C), while dbcAMP, a PKA activator, increased significantly the ATPase activity (Fig 5D). However, this increase disappeared in presence of indomethacin, a blocker of PGE2 synthesis (Fig 5E).

**Fig 6:**
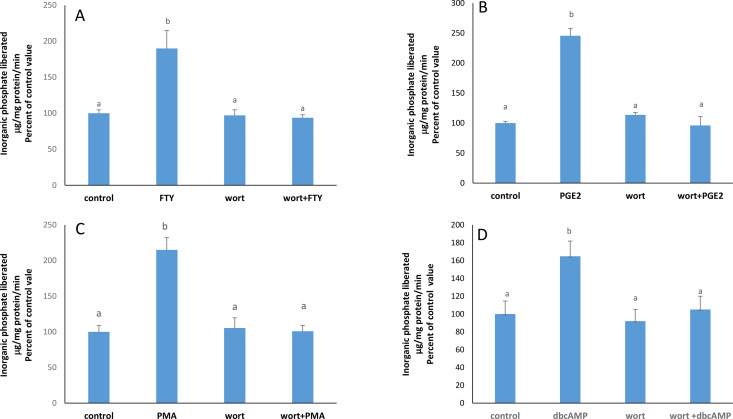
PI3K is downstream of FTY720P, PGE2, PKC, and PKA. A) Effect of FTY720P on the Na + /K + ATPase activity in the presence of wortmannin (100 nM) at 2 hours. The results are means ± SEM. N = 3. One way ANOVA was conducted to test for statistical significance (F = 9.103; p = 0.0119) followed by a Tukey-Kramer test. Bars not sharing a common superscript are significantly different from each other at p < 0.01.**B)** Effect of PGE2(1nM) on Na + /K + ATPase activity in the presence of wortmannin(100nM) at 2 hours. The results are means ± SEM. N = 3. Statistical significance was tested by a one-way ANOVA (F = 50.46; p = 0.0001) followed by a Tukey-Kramer test. Bars not sharing a common superscript are significantly different from each other at p < 0.05. **C)** Effect of PMA (100 nM) in the presence of wortmannin (100 nM) on the activity of the Na + /K + ATPase at 2 hours. The results are means ± SEM. N = 3. Statistical significance was tested by a one-way ANOVA (F = 18.86; p = 0.0006) followed by a Tukey-Kramer test. Bars not sharing a common superscript are significantly different from each other at p < 0.01. **D)** Effect of dbcAMP (10 µM) on the activity of the Na + /K + ATPase in the presence of wortmannin (100 nM) at 2 hours. The results are means ± SEM. N = 3. Statistical significance was tested by a one-way ANOVA (F = 4.593; p < 0.037) followed by a Tukey-Kramer test. Bars not sharing a common superscript are significantly different from each other at p < 0.01.

In presence of wortmannin, a PI3K inhibitor, the stimulatory effect of each of FTY720P (Fig 6A), PGE2 (Fig 6B), PMA (Fig 6C), and dbcAMP (Fig 6D) disappeared.

### PGE2, PI3K and PKA inhibit ERK

**Fig 7:**
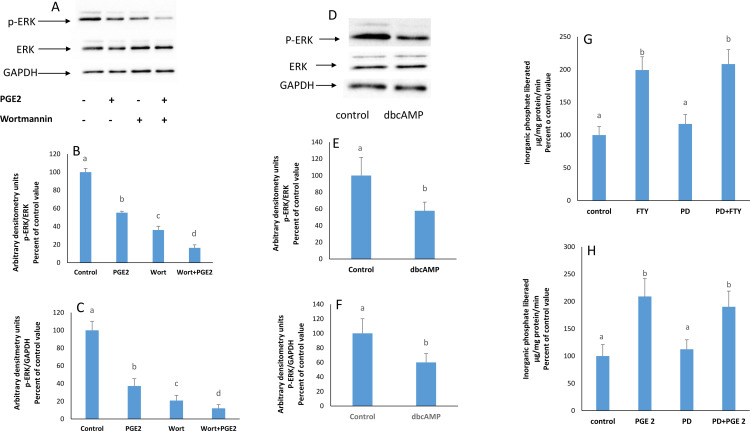
ERK is not along the pathway. A) Effect of PGE2 and wortmannin on the protein expression of phosphorylated ERK at 2 hours. The blots are representative of an experiment repeated 3 times. Values were normalized to **B)** ERK or **C)** GAPDH and reported as arbitrary densitometry units. Bars not sharing a common superscript are significantly different from each other at p < 0.01. Significant differences were tested by ANOVA [(B): F = 14.03; p = 0.0003 – (C): F = 121.6; p = 0.0001] followed by a Tukey-Kramer test.**D)** Effect of dbcAMP on the protein expression of phosphorylated ERK at 2 hours. The blots are representative of an experiment repeated 3 times. Values were normalized to **E)** ERK or **F)** GAPDH and reported as arbitrary densitometry units. Bars not sharing a common superscript are significantly different from each other. Significant differences were tested by Student’s t-test [(E) p = 0.0301; t = 5 – (D) p = 0.034; t = 5.2]. **G)** Effect of FTY720P (7.5 nM) on the activity of the Na + /K + ATPase in the presence of PD98059 (50 µM) at 2 hours. The results are means ± SEM. N = 3. Statistical significance was tested by a one-way ANOVA (F = 8.66; p = 0.0094) followed by a Tukey-Kramer test. Bars not sharing a common superscript are significantly different from each other at p < 0.01. **H)** Effect of PGE2 (1 nM) on the activity of the Na + /K + ATPase in the presence of PD98059 (50 µM) at 2 hours. The results are means ± SEM. N = 3. Statistical significance was tested by a one-way ANOVA (F = 8.143; p = 0.0155) followed by a Tukey-Kramer test. Bars not sharing a common superscript are significantly different from each other at p < 0.01.

PGE2 was also reported to signal via the extra-cellular -regulated kinase (ERK) [17]. The kinase is activated by phosphorylation. Therefore, western blot analysis was performed to probe for its phosphorylated form and to check for its involvement in the signaling pathway. Cells treated individually with PGE2 or with the PI3K inhibitor wortmannin showed a reduced expression of p-ERK which was reduced even further in their simultaneous presence (Fig 7A).

The PKA activator dbcAMP also reduced the protein expression of p-ERK (Fig 7D).

### ERK is not a mediator of the FTY720P/PGE2 -induced Na^+^/K^+^ ATPase stimulation

Since PGE2, PI3K, and PKA, were found to modulate ERK activity, the involvement of ERK in the pathway was checked. In the presence of PD98059, an inhibitor of ERK, the stimulatory effect of FTY720P and PGE2 on the Na^+^/K^+^ ATPase was still observed (Fig 7G & 7H).

## Discussion

The current results revealed that in Caco-2 cells FTY720P exerts opposite effects on the Na + /K + ATPase at different time points with a maximal inhibitory effect observed at 15 min and a maximal stimulatory effect appearing at 90 min and maintained till 3hrs. The early inhibition is expected to reduce sodium and water absorption and leads to water loss. The switch from inhibition to stimulation may be an attempt to restore water balance. The inhibition was shown in a previous work to be mediated via S1PR2 and PGE2. This work focused mainly on the 2hr- effect.

### FTY720P acts via S1PR2

At 2 hours, FTY720P increased significantly the activity of the Na^+^/K^+^ ATPase. Being a S1P analogue, FY720P is expected to act via five different receptors: S1PR1, S1PR2, S1PR3, S1PR4, and S1PR5. We previously found that S1PR2 and S1PR3 are the major receptor types expressed in Caco-2 cells, with S1PR2 having the highest expression [11]. Therefore, we focused mainly on these two types. The FTY720P-induced increase in the pump’s activity was still observed in presence of CAY 10444, a blocker of S1PR3 but disappeared completely in presence of JTE-013, a blocker of S1PR2, suggesting that FTY720P acts by binding only to S1PR2. Had there been another receptor involved in addition to S1PR2, some stimulatory activity would still have been observed. In further support of these findings came the results with CYM-5520 and CYM-5541 respective agonists of S1PR2 and S1PR3 which showed a significant increase in the ATPase activity by CYM-5520 while CYM-5541 was without any effect on the pump. Taken together, the results suggest that FTY720P acts at 2hrs via S1PR2.

The literature reports that FTY720P can bind to all S1P receptors except S1PR2. However, this study along with several recent papers showed that FTY720P can activate S1PR2 in many cell types, like myofibrobalsts, lung fibroblasts, and hepatocellular carcinoma cells [18,19].

### S1PR2 acts via Gq and PKC

S1PR2, like all S1PRs, is a G-protein coupled receptor. It may act via Gi/o, Gq, or G12/13 [20] with every G-protein promoting a specific signaling pathway involving different signaling molecules. Gi proteins inhibit adenylyl cyclase leading to a decrease in cAMP levels and a subsequent decrease in PKA activity [21]. Gq proteins are known to activate the PLC pathway whereby phospholipase C catalyzes the production of diacylglycerol (DAG) and inositol 1,4,5-triphosphate (IP3) from phosphatidylinositol 4,5-bisphosphate (PIP2) [22]. In turn, DAG activates PKC [23] while IP3 increases intracellular calcium concentration. Thus, PKC and calcium seemed to be potential mediators. Inhibiting PKC with calphostin C, abolished completely the stimulatory effect of FTY720P on the Na^+^/K^+^ ATPase and brought its activity back to control levels. Treating the cells with PMA, a PKC activator mimicked the effect of FTY720P on the pump confirming an involvement of PKC in the signaling pathway. Since PKC inhibition abolished completely the stimulatory effect of FTY720P and brought the ATPase activity back to control levels, it can be inferred that only Gq proteins are activated and no other different G-protein sub-types. The results are in line with the reported modulatory effect of PKC on the Na^+^/K^+^ ATPase which varies with the type of cells and the prevailing context. In COS-7 cells, activated PKC phosphorylated the α1 subunit of the Na^+^/K^+^ ATPase at Ser-16 increasing its affinity to sodium ions and consequently its activity [24]. In intestinal cells, PKC exerted an inhibitory effect on the ATPase when activated by insulin [25]. In rat proximal tubules the PKC effect on the pump was dependent on the oxygenation levels: it was stimulatory under high oxygen levels and inhibitory under hypoxic conditions [26].

Three types of PKC-isozymes exist: conventional, novel, and atypical [27]. The conventional type is the only one that is calcium-dependent. Consequently, the involvement of calcium ions was tested. The FTY720P-induced stimulation of the Na + /K + ATPase was not observed when a calcium ion chelator, BAPTA/AM, was added, indicating that calcium is a mediator and that PKC is of the conventional type. Similarly, in kidney proximal tubules, PMA was reported to exert its stimulatory effect on the Na^+^/K^+^ ATPase through PKC-β, which belongs to the conventional PKC family and is calcium-dependent.

The results support a pathway in which S1PR2s are activated leading to the activation of Gq and PLC, and Calcium release.

### PGE2 is another mediator of S1PR2 signaling

We previously demonstrated an induction of PGE2 synthesis by S1PR2 [19]. The COX enzymes, COX1 and COX2, catalyze the synthesis of PGE2 from arachidonic acid. COX1 is constitutively expressed in tissues while COX2 is inducible.

Inhibiting the COX enzymes with indomethacin abolished completely the stimulatory effect of FTY720P implying that S1PR2 increases the activity of the Na^+^/K^+^ ATPase via PGE2. Furthermore, exogenous PGE2 imitated the stimulatory effect of FTY720P on the ATPase, suggesting that FTY720P acts through S1PR2 to induce COX2 expression (COX-1 being constitutively active) and subsequent synthesis of PGE2 leading eventually to Na^+^/K^+^ ATPase activation.

Similar results correlating S1PR2 to PGE2 synthesis have been reported in the literature. In the Wilms’ tumor cell line (WiT49), S1P was shown to induce COX2 expression through S1PR2 leading to PGE2 production [28]. Moreover, in S1PR2 knockout mice, the cells of the retina displayed a subsided COX2 expression, suggesting a link between S1PR2 activation and PGE2 synthesis [18]. Also, in renal mesangial cells, S1P improved cell migratory ability by promoting COX2 expression through S1PR2 [29].

### Position of PGE2 with respect to PKC

The literature reports PKC as an upstream modulator of PGE2 synthesis [30]. In this work, PMA was unable to enhance the activity of the ATPase when PGE2 synthesis was blocked with indomethacin, inferring that PKC is indeed upstream of PGE2 along the pathway. The stimulatory effect of exogenous PGE2 appeared unaltered in presence of a PKC inhibitor (calphostin C), confirming the upstream position of PKC with respect to PGE2.

These results are in line with other works reporting a PKC-induced synthesis of PGE2 [31,32].

### NF-KB is needed for COX-2 transcription and PGE2 production

NF-κB has been reported as a transcription factor needed for COX2 transcription [33] and hence it is suspected to be along the signaling pathway. The inhibition of NF-κB completely abolished the stimulatory effect of FTY720P on the ATPase confirming its essential role in PGE2 production. Such a role has been reported by other works [34,35].

On the other hand, NF-KB was shown to be targeted by PKC [36,37] and by S1P through activation of S1PR2, Gq and the PLC pathway [38].

The data indicate so far that activation of S1PR2 activates PKC which in turn activates NF-κB leading to PGE2 release. To rule out the possibility that NF-κB may be enhancing the expression of another mediator downstream of PGE2, Caco-2 cells were incubated with PGE2 in the presence and absence of NF-κB inhibitor. Inhibiting NF-κB did not alter in any way, the stimulatory effect of PGE2 on the pump suggesting that the transcription factor acts upstream of PGE2.

### PGE2 signaling

PGE2 acts via four different EP receptors (EP1–4), all of which are coupled to G-proteins. To identify the ones responsible for inducing the stimulatory effect on the Na^+^/K^+^ ATPase, each EP receptor was tested at a time.

EP1 couples to Gq [39] leading to PLC and PKC activation. Blocking EP1 receptors with SC-1922 or inhibiting PKC with calphostin C, did not affect in any way the PGE2-induced stimulation of the Na^+^/K^+^ ATPase suggesting that PGE2 does not act via EP1. The activity of the Na^+^/K^+^ pump in Caco-2 cells treated with Sulprostone, an EP-1 and EP-3 agonist, was similar to the control value indicating that neither EP1 nor EP3 are involved in the signaling cascade.On the other hand, EP2 and EP4 receptors are coupled to the Gs protein which is known to activate PKA by increasing cAMP synthesis [40]. Upon inhibition of PKA with RpcAMP, the stimulatory effect of PGE2 was completely abrogated thus indicating that PKA lays downstream PGE2 along the pathway and suggesting a potential involvement of EP2 or/and EP4. However, PGE2 could still increase the activity of the ATPase in presence of PF-04418948, a blocker of EP2 receptors, and treating Caco-2 cells with Butaprost, an EP2 agonist, did not affect the pump’s activity inferring that EP2 receptors are not involved.

EP4 receptors may couple also to Gs or Gi [40]. Blocking these receptors with BGC-20–1531 HCL abolished completely the PGE2-induced stimulation of the pump confirming that the prostaglandin acts via EP4 that are coupled to Gs.

As for EP3, it is known to be coupled to Gi proteins which inhibit PKA. If PGE2 acts via PKA inhibition, then a similar stimulatory effect on the ATPase should be observed when PKA is inhibited with RpcAMP. Since this was not the case, the involvement of EP3 receptors was ruled out. Indeed, the stimulatory effect of PGE2 was still observed in presence of L798601, a blocker of EP3 receptors, and treating Caco-2 cells with Sulprostone, an EP3 agonist, did not alter the Na^+^/K^+^ ATPase activity suggesting that EP3 receptors are not involved in the stimulatory pathway elicited by PGE2.

It can be concluded that PGE2 activates EP4 receptors which are coupled to Gs leading to PKA activation.

### PI3K as a possible mediator

S1Pand PGE2 may signal by modulating the activity of PI3K [41,42]. PI3K, in turn, is known to regulate the activity of Na^+^/K^+^ ATPase. Therefore, PI3K was suspected to mediate the action of FTY720P on the ATPase. Inhibiting PI3K with wortmannin prevented FTY720P from exerting its stimulatory effect, indicating that PI3K kinase is an essential mediator. Modulation of Na^+^/K^+^ ATPase by PI3K has been reported in several papers and the effect of the kinase seems to be tissue-dependent [11,43].

The inhibition of PI3K with wortmannin completely prevented PMA, a PKC activator, as well as PGE2 from exerting their stimulatory effect on the Na^+^/K^+^ ATPase, implying that PKC and PGE2 are upstream PI3K. Such an effect of the prostaglandin on PI3K is supported by many studies conducted in various tissues [42].

Two mediators have been identified downstream PGE2 so far: PKA and PI3K.

Since EP4 receptors couple to Gs, PKA should be the first mediator of Gs and upstream PI3K. Inhibiting PI3K with wortmannin abrogated completely the effect of dbcAMP on the pump supporting the upstream position of PKA with respect to PI3K. The results are in line with many studies reported in the literature [44].

The extracellular- regulated kinase (ERK) is a well-recognized downstream effector of PGE2 and PI3K [45] and is phosphorylated upon activation. Western blot analysis revealed that the expression of p-ERK was reduced when PI3K was inhibited with wortmannin, suggesting that PI3K activates ERK. Similarly, treating Caco-2 cells with PGE2 led to a decrease in the expression level of p-ERK that was reduced further when PI3K was inhibited simultaneously with wortmannin indicating that PGE2 inhibits ERK while PI3K activates it. The results suggest the presence of two distinct pathways affecting ERK activity. One pathway activates ERK via PI3K and the other inhibits ERK through mediators that still need to be determined. The inhibitory pathway is dominant over the stimulatory pathway, and this is why the net effect of PGE2 on ERK is inhibitory.

PKA has also been reported as an upstream effector of ERK [45] and since it was shown to be downstream PGE2 in the FTY720P-induced signaling pathway it may modulate ERK activity. Treating Caco-2 cells with the PKA activator dbcAMP resulted in a significant reduction in ERK phosphorylation thus affirming the inhibitory effect of PKA on ERK. The results imply that PKA which is activated by PGE2 lies along the inhibitory pathway initiated by PGE2 and inhibits ERK.

Although the data reveal a modulation of ERK activity by PGE2, PI3K, and PKA which are all downstream effectors of FTY720P, yet ERK does not seem to be along the signaling pathway leading to a stimulation of Na + /K + ATPase, since upon inhibition of ERK with PD98059, FTY720P was still capable of inducing the same increase in the ATPase activity. This ERK inhibition paradox suggests pathway branching. PGE₂ may simultaneously regulate ATPase activity via PKA/PI3K and inflammation or another cellular function via ERK inhibition. Future work on single-cell RNA-seq could identify distinct cellular subpopulations mediating these effects.

## Conclusions

In conclusion, at 2 hours, FTY720P increases the activity of Na^+^/K^+^ ATPase by binding to S1PR2s and activating Gq proteins leading to a stimulation of PLC and PKC. The latter induces the direct or indirect phosphorylation and the subsequent ubiquitinylation and degradation of IκB, thus removing the inhibition from the transcription factor and causing its translocation to the nucleus where it induces the transcription of the COX2 enzyme. COX-2, then, catalyzes the synthesis of PGE2 which binds to EP4 receptors activating Gs proteins. Gs proteins in turn, activate adenylyl cyclase increasing cAMP levels and stimulating sequentially PKA, PI3K, and the Na^+^/K^+^ ATPase. Although ERK activity is modulated by PKA and PI3K yet it is not along the signaling pathway leading to the pump activation.

We demonstrated in a previous work an early inhibitory effect of FTY720P on the Na^+^/K^+^ ATPase that peaked at 15 min and was mediated via S1PR2 and PGE2, with the latter acting via the Gi-coupled EP3 receptors causing a decrease in cAMP levels and PKA activity. The opposite stimulatory effect observed at 2hrs is still mediated via S1PR2s and leads to PGE2 release, but the prostaglandin in this case acts through the Gs-coupled EP4 receptors which activate PKA. The opposite effects on the pump may be ascribed to the opposite effect on PKA.

Why different EP receptors are involved at 15 min and 2 hours?

The βγ subunits released upon activation of EP3 activate G protein-coupled receptor kinase which induces desensitization of the receptors [46]. Switching of the coupling of the GPCRs to different G proteins has been reported before. In the case of the beta2-adrenergic receptor the switch from Gs to Gi was dependent on PKA. The phosphorylation of the receptor by PKA reduces its affinity for Gs and increases its affinity for Gi [47]. Drawing a parallel scenario for EP3 and EP4 receptors, one could propose that the desensitization of EP3 receptors lifts the inhibition on adenylate cyclase, leading to an increase in cAMP levels. This rise in cAMP activates protein kinase A (PKA), which subsequently phosphorylates EP4, promoting its coupling to Gs proteins. Real-time monitoring of G-protein activation (e.g., BRET biosensors) could be used in the future work to test this hypothesis and resolve this temporal regulation.

**Fig 8:**
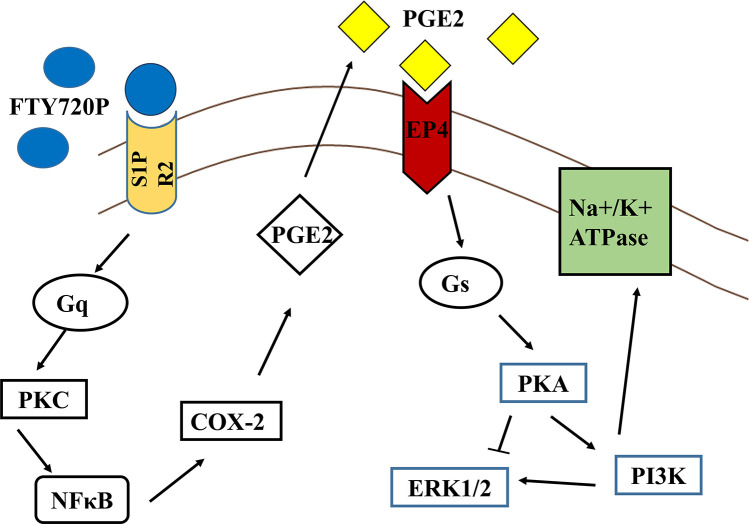
The signaling pathway mediating the stimulatory effect of S1P on the Na^+^/K^+^ATPase.

Fig 8 is a scheme summarizing this signaling pathway.

Our findings are clinical pertinent. By uncovering the mechanistic basis of dysregulated S1P/PGE₂ signaling in IBD, our study provides critical insight into disease pathophysiology. Therapeutic modulation of S1PR2 or EP4 may enable correction of electrolyte imbalance without aggravating inflammation [48], offering a novel strategy for medical intervention. In addition since calcium-dependency suggests conventional PKCs (e.g., PKCβ), while PI3K’s role may involve p110α/p110δ isoforms. Isoform-selective inhibitors (e.g., A66 for p110α) could refine therapeutic targeting.

## Supporting information

S1 data set Raw values for Figs 1–7.
(XLSX)

S2 Fig Original images corresponding to Figs 7A & 7D.
(PDF)
