# Supplementary material for: Mediators of the stimulatory effect of S1P on colonic Na+/K+ ATPase
Source: PLoS One. 2025 Aug 26;20(8):e0330818. doi: 10.1371/journal.pone.0330818 (PMC12380338; doi:10.1371/journal.pone.0330818)

Total ERK

Fig 7A

Effect of PGE2 in presence and absence of wortmannin on the expression of total ERK

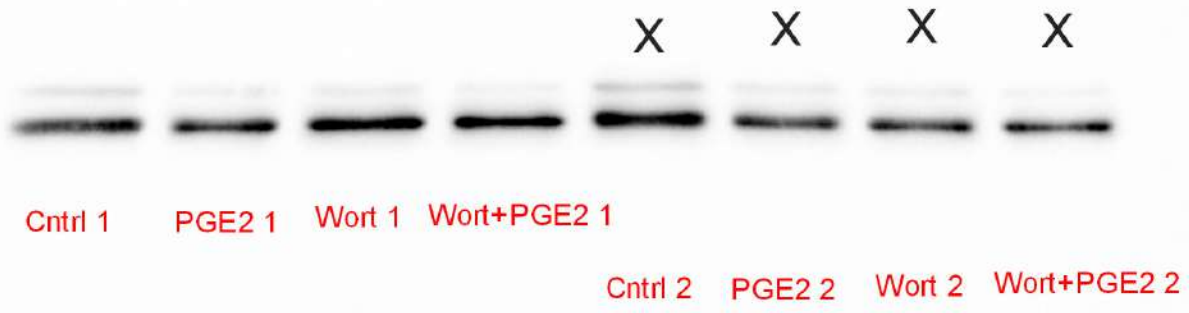

Fig 7A

GAPDH

GAPDH expression in cells treated with PGE2 in presence and absence of wortmanin

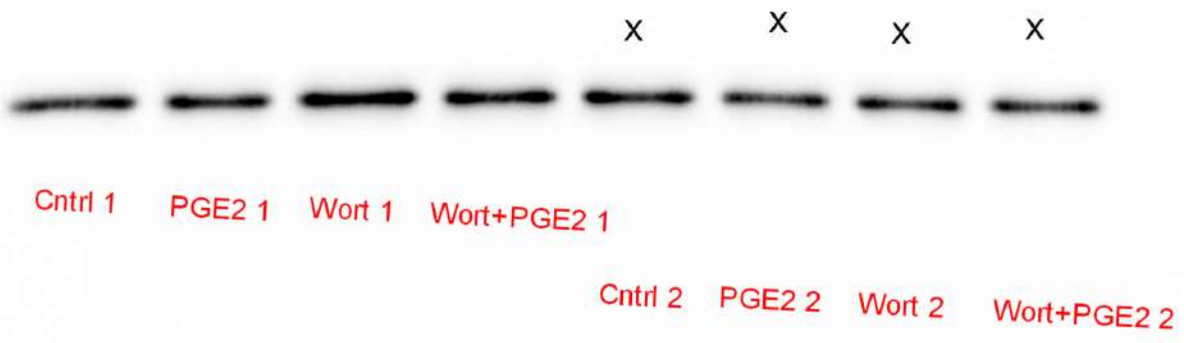

p-ERK

Fig 7A

Effect of PGE2 in presence of wortmannin on the expressio of pERK

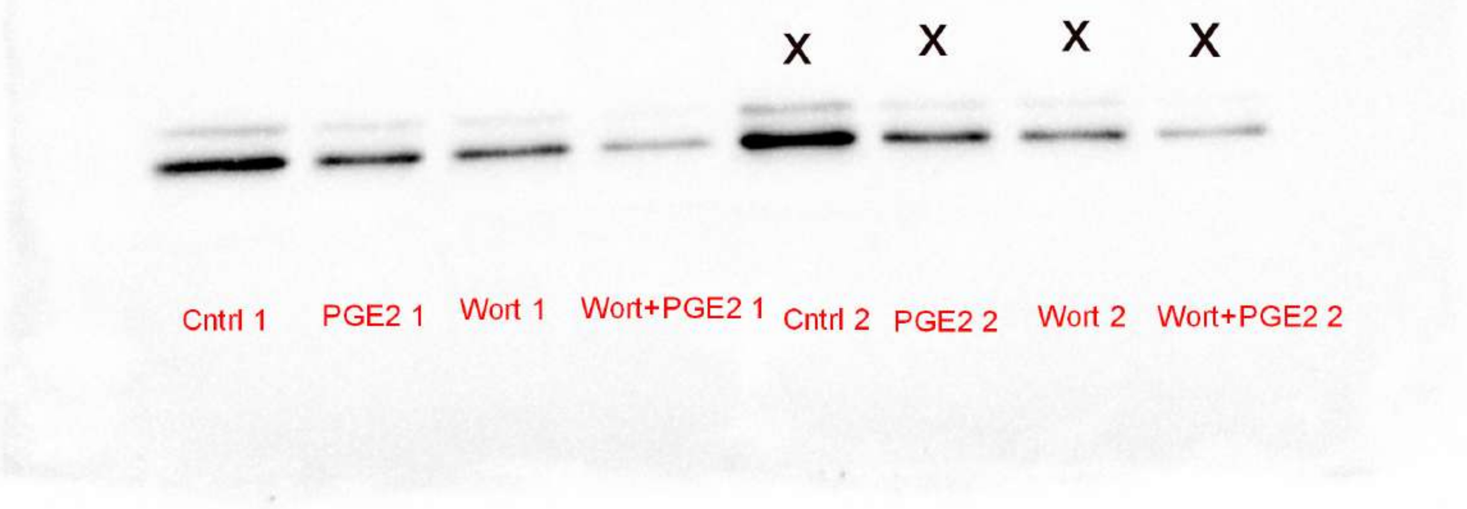

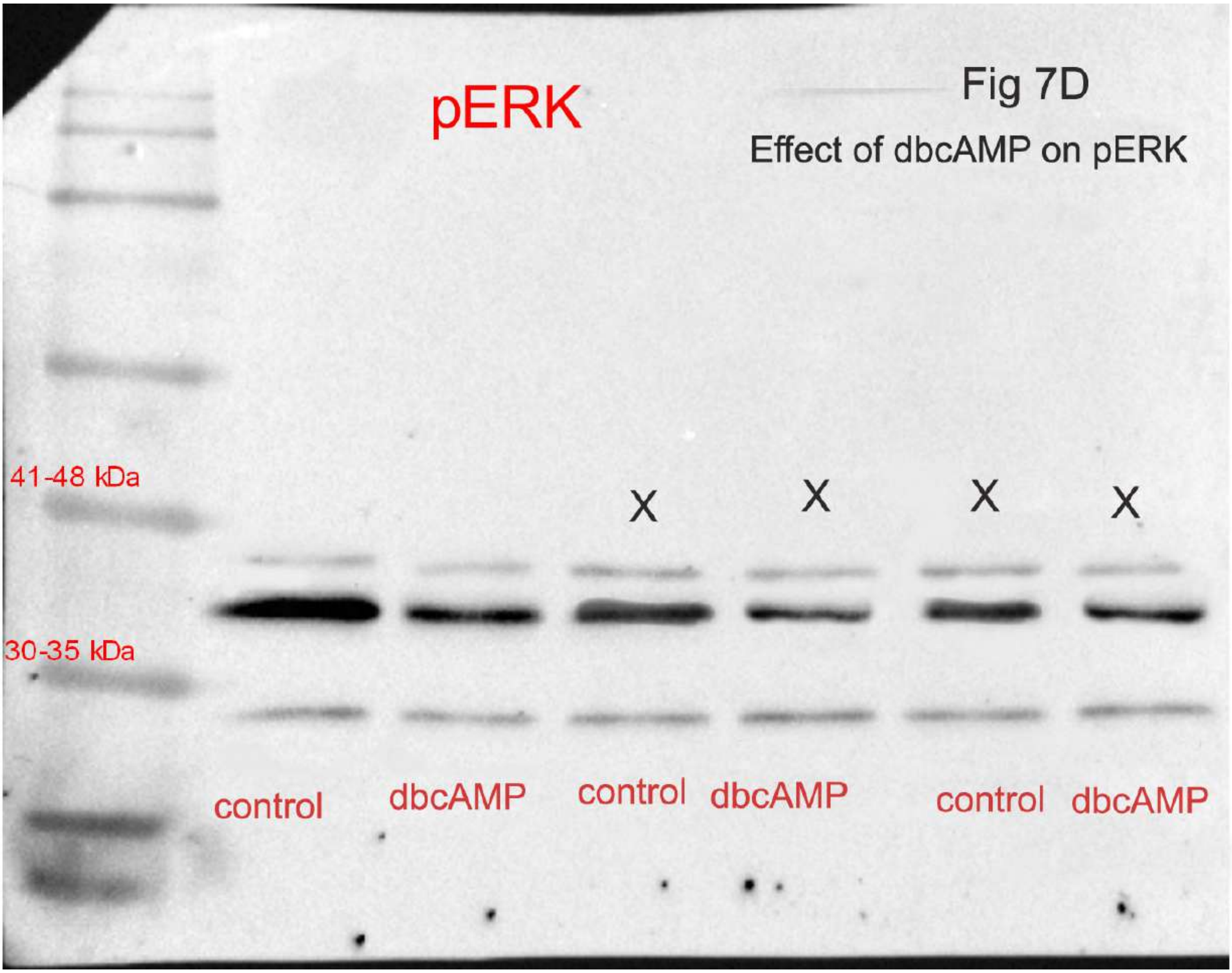
pERK
Fig 7D
Effect of dbcAMP on pERK
41-48 kDa
30-35 kDa
X
X
X
X
control
dbcAMP
control
dbcAMP
control
dbcAMP

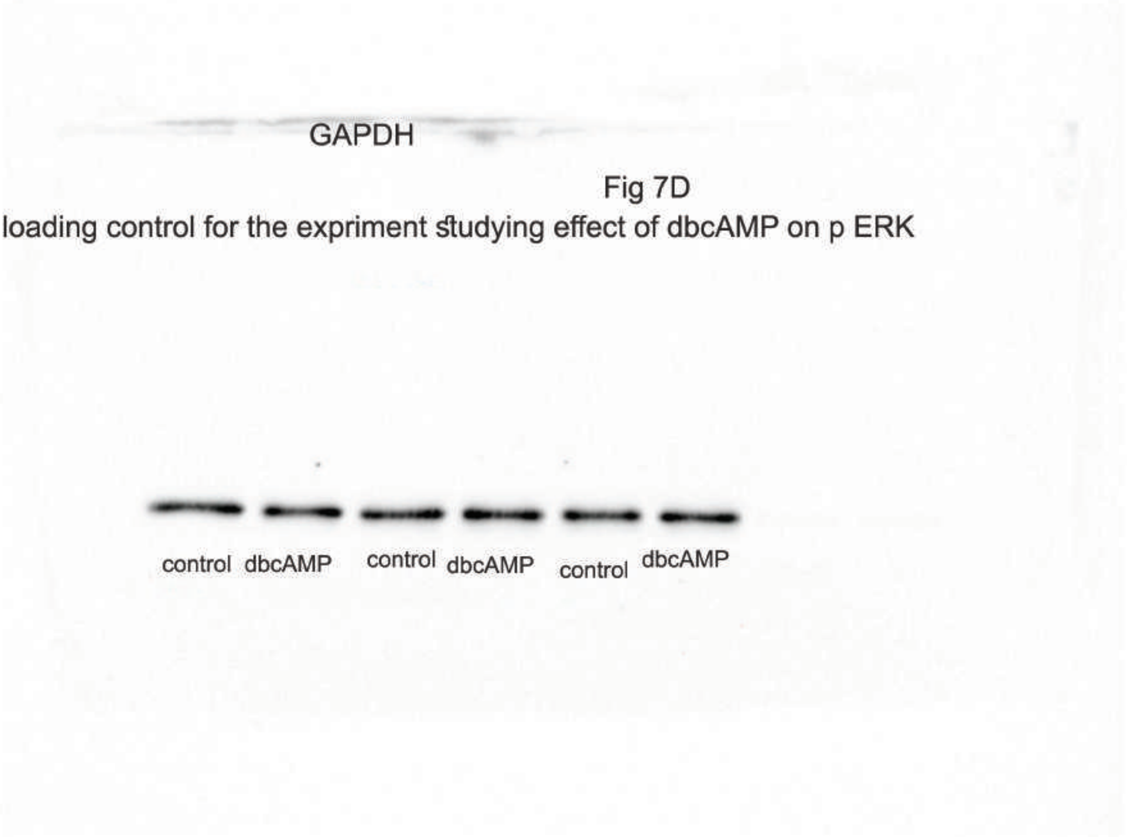

Fig 7D

loading control for the expriment studying effect of dbcAMP on p ERK

Fig 7D

Effect of dbcAMP on the expression of total ERK

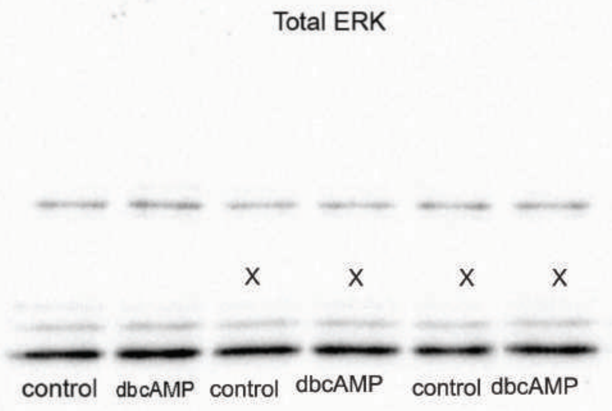

Supplement: S2 Fig — Original images corresponding to Figs 7A & 7D. (PDF) [file pone.0330818.s002.pdf]
